# A realist review to understand the complexity of effective management of type 2 diabetes and hypertension

**DOI:** 10.3389/phrs.2026.1608655

**Published:** 2026-06-01

**Authors:** Fatemeh Ehteshami, Anna Verjans, Rachel Cassidy, Fabrizio Tediosi, Günther Fink, Daniel Cobos Muñoz

**Affiliations:** 1 Swiss Tropical and Public Health Institute (Swiss TPH), Allschwil, Switzerland; 2 University of Basel, Basel, Switzerland; 3 KPM Center for Public Management, University of Bern, Bern, Switzerland; 4 Swiss Institute for Translational and Entrepreneurial Medicine, Bern, Switzerland; 5 University of Milan, Milan, Switzerland

**Keywords:** hypertension (HTN), low and middle income countries (LMICs), realist review, systems thinking, type 2 diabetes mellitus

## Abstract

**Objectives:**

We aimed to disentangle the mechanisms that govern the effective management of diabetes and/or hypertension in various contexts in low- and middle-income countries.

**Methods:**

We conducted a realist review guided by the Realist and MEta-narrative Evidence Syntheses Evolving Standards. We systematically searched electronic databases to examine in what contexts, how, and for whom programs targeting diabetes and/or hypertension care work through context-mechanism-outcome (CMO) configurations and then revised an initial program theory to a refined program theory.

**Results:**

We identified four CMO configurations. (1) Cross-sectoral partnerships activate the mechanisms of resource pooling and mobilization, improving healthcare services coverage and fostering sustainable local buy-in (2) Integration of disease-specific programs facilitates coordinated care, resulting in treatment adherence (3) Digitalized infrastructure and literacy enable the mechanisms of mHealth and eHealth implementation and clinical decentralization, improving access, coordination, and treatment adherence (4) Patient-centered care triggers the mechanisms of patient-provider communication and personalized care, leading to patient engagement and health education.

**Conclusion:**

This review provides insights into the relevance of contexts and their associated mechanisms for operationalizing successful models of care for diabetes and/or hypertension that enhance health outcomes.

## Introduction

The global prevalence of Diabetes Mellitus (DM) and Hypertension (HTN) has increased over the past few decades [1, 2], yet effective management of these conditions remains suboptimal in many settings, particularly in low- and middle-income countries [3, 4]. A cross-sectional study analyzing the results of nationally representative surveys determined that the total unmet need for DM care in 28 LMICs is estimated to be 77%, which is the sum of cases that are undetected, detected but undiagnosed, diagnosed but untreated, and treated but not controlled. The unmet need and low rate of control (22.8%) indicate a significant gap in DM care in LMICs [5]. A further study of the care cascade for HTN in low-income settings found only 16% of hypertensive patients had achieved control, with 48% of people with HTN being aware of their condition and 35% receiving treatment [6]. Poor control of DM and HTN accelerates their progression to complicated conditions such as cardiovascular disease [7–9]. Therefore, effective management of patients living with these conditions is crucial.

The required continuous and coordinated care and the presence of comorbidities with DM and HTN further complicate effective management. Common comorbidities of diabetic patients include peripheral vascular disease, end-stage renal disease, blindness, and amputations [9]. HTN is also the primary risk factor contributing to the development of atherosclerotic vascular diseases such as cardiac, cerebrovascular, and renal conditions [10]. There are other comorbidities with DM and/or HTN, such as depression [11, 12], obesity [13], DM and tuberculosis [14], and HIV/AIDS [15].

Due to the complications that arise from ineffective DM and HTN management, it is critical that we identify and understand the complex and multifaceted pathways for effective DM and HTN care. Previous reviews identified several key factors of an effective model of care that contribute to the management of DM and/or HTN in LMICs, such as quality communication among providers and patients, the availability of essential medicines, trained health professionals at decentralized levels, coordination between providers, standardization of clinical guidelines, community and peer engagement, and the integration of technologies into disease management [16, 17]. Despite these insights, there remains a need for research that explores the causal linkages among these factors and examines the underlying contexts and mechanisms that influence the management of these conditions.

A realist review evidentiary approach was appropriate for this study as it is used to unravel how a program works (or does not work) by addressing what works, for whom, and in what circumstances through Context, Mechanism, Outcome (CMO) configurations [18]. We conducted a realist review to understand how, in what contexts, and for whom multicomponent programs targeting DM and/or HTN management are effective - in other words, to identify mechanisms of DM and/or HTN management by which these programs lead to specific health outcomes across various contexts.

## Methods

### Study design

This realist review followed the Realist and MEta-narrative Evidence Syntheses Evolving Standards (RAMESES), guided by a checklist (Supplementary Data Sheet 1) [19]. The study protocol was published in PROSPERO (registration code: CRD42023445454), with further rationale and the methodological approach defined in a peer-reviewed protocol [20]. This review used existing published literature; therefore, ethical approval was not required. The study phases were conducted according to Pawson et al.’s methodology for a realist review [18]. A realist review is characterized by several key components, namely: an initial program theory, context, mechanism, outcome (CMO) configuration, and a refined program theory [21] Table 1.

**TABLE 1:** Components of realist terms in the review (A realist review to understand the complexity of effective management of type 2 diabetes and hypertension, 2013–2023) (low- and middle-income countries).

Initial program theory	A combination of factors that conceptualize how DM and/or HTN management in LMICs work. A CLD based on a preliminary review of evidence serves as an initial program theory in this review
Context	Any state that affects how a mechanism is implemented. It might facilitate, activate, or deactivate a mechanism
Mechanism	A process triggered and generated in specific contexts that leads to health outcomes
Outcome	A result of the interaction of mechanisms under a specific context
Refined program theory	Identified CMO configurations update and refine the initial program theory to a refined program theory

#### First phase: conceptualization of the initial program theory to address the complexity of DM and HTN management in LMICs using a CLD

W adopted a systems thinking approach, specifically the use of a causal loop diagram (CLD) to unpack the complexity of DM and/or HTN management and understand which underlying factors influence the (in)effective management of these conditions. Systems thinking is a way to understand complex, dynamic, and interconnected systems by emphasizing relationships rather than isolated components [22, 23]. Through a range of tools and techniques, systems thinking approaches seek to identify interconnections among system elements and processes [24]. CLDs are one systems thinking tools that offer valuable insights to key actors by illustrating the interactions between system components and the causal linkages involved in addressing a problem [25].

In this phase, we developed a CLD as a problem-driven tool and representation of the initial program theory. To this end, we conducted a preliminary search and reviewed articles, including qualitative studies, mixed-method studies, and reviews. We particularly delved into qualitative case studies in LMICs, which offered opportunities to delineate the causal pathways and inform the CLD development according to the perceptions of healthcare providers and patients with DM and/or HTN. We extracted the healthcare provider-related factors, such as availability, accessibility, and knowledge of healthcare workers, and patient-related factors, such as awareness and knowledge, treatment adherence, care-seeking behavior, and financial constraints, and then mapped them through a CLD. This phase of work is completed and published elsewhere [20].

#### Second phase: search, selection, appraisal, and extraction of evidence regarding programs for DM and/or HTN management in LMICs

In this phase, we developed inclusion/exclusion criteria and search strategies and conducted systematic literature searches. We then assessed the quality of the included eligible studies and extracted the data to identify CMO configurations within programs targeting healthcare services for the management of DM and/or HTN.

##### Eligibility criteria

The list of inclusion and exclusion criteria is shown in Table 2. We included articles in English published between 2013 and 2023 describing healthcare services related to the management of Type 2 Diabetes Mellitus (T2DM) and/or HTN in LMICs. The management of T2DM and HTN was defined as approaches that enable achieving and maintaining glycemic control and blood pressure levels, preventing or delaying complications, and enhancing quality of life [26, 27]. The list of countries defined as LMICs followed the World Bank classification of *per capita* gross national income in 2021 [28]. Studies describing the management of Type 1 Diabetes Mellitus (T1DM), gestational, monogenetic, or secondary DM were excluded as different approaches should be adopted for various types of DM [29]. All studies had to meet all inclusion criteria.

**TABLE 2:** Eligibility criteria for literature review (A realist review to understand the complexity of effective management of type 2 diabetes and hypertension, 2013–2023) (low- and middle-income countries).

Criteria	Inclusion	Exclusion
Type of study	Studies that incorporated at least two elements of the WHO building blocks other than healthcare services delivery [30]	Studies without the description of programs and mechanisms, and without analysis of control of DM and/or HTN
	All types of studies that described the implementation of programs and healthcare services related to the management of T2DM and/or HTN	
	Studies that analyzed the outcomes related to clinical control of DM and/or HTN	
Countries	LMICs	HICs
Setting	Primary healthcare facilities	Other settings
Language	English	Other languages
Publication year	2013–2023	Other years

##### Search strategy, study selection, and data extraction

We conducted a literature search in four electronic databases, namely CENTRAL, Web of Science, PubMed, Embase, and supplemented these with additional records from a manual search and reference lists of reviewed publications. We developed the search strategy using a combination of medical subject headings (MeSH) and free text, including terms related to “healthcare services”, “intervention” “program”, “care model”, “type 2 diabetes”, “hypertension”, and their synonyms and variant terms as shown in Supplementary Data Sheet 2.

**FIGURE 1:**
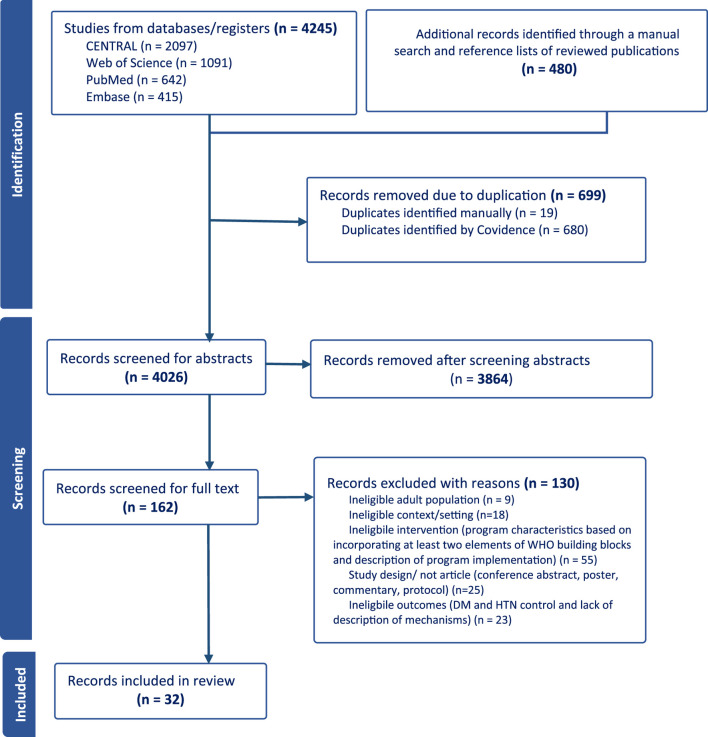
Overview of study screening and inclusion (A realist review to understand the complexity of effective management of type 2 diabetes and hypertension, 2013–2023) (low- and middle-income countries).

The screening process for the review is shown in detail in Figure 1. We imported all evidence into EndNote and Covidence as a tool for screening titles/abstracts and full texts. Two independent authors (FE and AV) screened full texts to assess eligibility. We recorded reasons for exclusion and resolved any disagreements through discussion to reach a consensus. In case they could not reach a consensus, the third author DC reviewed the study. We extracted key characteristics of the eligible studies, including study design, year of publication, setting, key contexts, mechanisms, and outcomes, which are summarized in Supplementary Data Sheet 3.

##### Assessment of study quality and publication bias

We assessed study quality and risk of bias with the Mixed Methods Appraisal Tool (MMAT) version 2018 [31]. Two reviewers (FE and AV) were involved in the quality assessment, and the third author DC was involved in solving any disagreements. The quality criteria of the MMAT tool were assessed by responding “Yes”, “No”, or “Can’t tell” referring to whether the study did or did not meet each criterion. The first two criteria are independent of the study design, whereas the remaining five are defined according to the type of study, as shown in Supplementary Data Sheet 4.

#### Third phase: analysis of data, development of CMO configurations, and program theory refinement

The eligible studies were scrutinized to determine the CMO configurations for the management of DM and/or HTN. In a realist approach, data are not only limited to results and measured outcomes of a study but also contain author descriptions and discussions that serve as a rich and valuable data source that makes clear and explicit whether and how a program works [32]. Accordingly, the full texts of the included studies were reviewed to become familiar with the various programs implemented across different countries. Next, the descriptions of the programs and author explanations within the included studies (such as the design and implementation pathways, the components involved, how they were delivered, and the target population) were highlighted. The relevant information was extracted and compiled into a spreadsheet in a Word document, including the title, year of the study publication, country, study design, description of the program and health outcomes, and clinical effectiveness of DM and HTN. We then identified prominent patterns of contexts, mechanisms, and outcomes through the iterative analysis process by the research team.

Furthermore, the CLD was updated and revised according to the identified CMO configurations. We presented the causal links of the CLD by arrows and polarities (+/−) that show the relationship between two variables in which an increase or decrease in one variable influences the other variable in a similar (+) or opposite (−) way. A series of causal links created two key loop behaviors, either a positive (reinforcing) or a negative (balancing) loop. A balancing loop is self-correcting, as the effects are dampened throughout the loop. A reinforcing loop is self-reinforcing, as the effects are amplified throughout the loop, which may result in a virtuous cycle (desirable effects) or a vicious cycle (negative effects) [33, 34].

## Results

The initial search yielded 4,725 articles. We excluded 3864 studies after a preliminary screening process, resulting in 162 articles that were eligible for full-text screening. A total of 32 studies met all the inclusion criteria (see Figure 1). Key characteristics of the included studies are shown in Table 3. One study included six middle-income countries, along with two high-income countries [35]. The geographical distribution and the number of included studies in each country are shown in Supplementary Data Sheet 5.

**TABLE 3:** Key characteristics of the included studies (A realist review to understand the complexity of effective management of type 2 diabetes and hypertension, 2013–2023) (low- and middle-income countries).

Authors/References	Region(s)	Publication year	condition under study
Lim et al. [35]	8 Asia-Pacific countries (India, Indonesia, Malaysia, the Philippines, Singapore, Taiwan, Thailand, and Vietnam)	2021	DM
Boch et al. [36]	Mongolia, Senegal, Brazil	2022	HTN
Ali et al. [37]	India and Pakistan	2016	DM
Jafar et al. [38]	Bangladesh, Pakistan, and Sri Lanka	2016	HTN
Hickey et al. [39]	Kenya and Uganda	2021	HTN
Lou Q et al. [40]	China	2020	DM
Zhu et al. [41]	China	2018	HTN
Wang et al. [42]	China	2021	HTN and/or DM and COPD
Jia et al. [43]	China	2021	DM
Sun et al. [44]	China	2022	HTN
Zhou et al. [45]	China	2022	HTN
Ajay et al. [46]	India	2016	DM and HTN
Ali et al. [47]	India	2020	DM and depression
Prabhakaran et al. [48]	India	2019	HTN and DM
Ramli et al. [49]	Malaysia	2016	DM
Low et al. [50]	Malaysia	2013	HTN
Paluyo et al. [51]	Philippines	2021	DM and HTN
Pilleron et al. [52]	Philippines	2014	DM
Khan et al. [53]	Pakistan	2018	HTN
Xie et al. [54]	Bangladesh	2023	DM and HTN
Chan et al. [55]	Kazakhstan	2020	DM and HTN
Valdes Gonzalez et al. [56]	Cuba	2020	HTN
Dethlefs et al. [57]	Dominican Republic	2019	DM and HTN
Adler et al. [58]	Ghana	2019	HTN
Collins et al. [59]	Tajikistan	2019	HTN
Kingue et al. [60]	Cameroon	2013	HTN
Zou et al. [61]	Sierra Leone	2020	DM and HTN
Morelli et al. [62]	Argentina	2023	DM
Doocy et al. [63]	Lebanon	2017	DM and HTN
Ameh et al. [64]	South Africa	2017	HTN, HIV
Patel et al. [65]	Malawi	2018	HTN and HIV
Birungi et al. [66]	Tanzania and Uganda	2021	DM, HTN and HIV

### Study quality

The MMAT results suggest that the included studies were generally of good quality (Supplementary Data Sheet 4). No study was excluded due to poor quality. For all studies, the first two questions regarding the clarity of the research questions and the relevance of the collected data to answer the research questions were selected “Yes”. In addition, three studies were program reports and could not be appraised using MMAT [51, 56, 65].

### Context, mechanism, outcome (CMO) configurations

The analysis of the included studies supported four CMO configurations to explain how contexts and mechanisms influence outcomes.

#### CMO1: cross-sectoral partnerships (C) pool and mobilize resources for the provision of healthcare services related to DM and HTN (M), improving healthcare services coverage and fostering sustainable local buy-in practices (O)

We found twelve studies conducted within the context of cross-sectoral partnerships. This allowed the implementation of mechanisms related to mobilizing and pooling resources for healthcare services that facilitated improved healthcare coverage and local buy-in [36, 40, 51–53, 55–59, 61, 65]. For example, a study in Ghana examined the Community-based Hypertension Improvement Project (comHIP), a public-private partnership (PPP) between the Ghana Health Service (GHS), FHI 360 (formerly Family Health International), and the Novartis Foundation. In the comHIP, FHI 360 and the Ministry of Health (MoH) conducted training for healthcare workers. The authors highlighted that comHIP’s strength lies in the local systems, such as the use of existing GHS protocols and medications, making it more sustainable compared to programs that depend on outside funding and resources. The outcomes of the implementation of comHIP showed that 72% (95% CI: 67%–77%) of participants had controlled blood pressure [58]. Another study evaluated the implementation of a program (CARDIO4Cities) in Ulaanbaatar in Mongolia, Dakar in Senegal, and São Paulo in Brazil. The selected cities represent diverse urban contexts across three continents; therefore, the program was tailored to address local priorities. It was co-designed between the Novartis Foundation and local authorities and public-private partners from various sectors. Mobilizing and engagement of non-traditional health players broadened opportunities for community outreach and HTN detection and awareness. The program was delivered based on contextual variations in health systems in each country. In Brazil, free treatment was available to all individuals according to local policy, whereas in other cities, the partnership worked with local authorities to incorporate HTN medicines into essential drug lists. This initiative, by engaging multiple partners and mobilizing non-health players, ensured local buy-in. The findings showed the proportion of enrolled patients who achieved HTN control while taking medication roughly tripled in São Paulo (from 12.3% to 31.2%) and Dakar (from 6.7% to 19.4%), and increased more than six-fold in Ulaanbaatar (from 3.1% to 19.7%) [36].

#### CMO2: integration of disease-specific programs for chronic conditions (C) enhances team-based coordinated care for multiple chronic comorbidities (M), improving treatment adherence (O)

Several studies indicated that integrating disease-specific programs for specific chronic conditions creates concurrent team-based coordinated care for multiple chronic comorbidities. The interactions of mechanisms within this context enhance treatment adherence [39, 42, 47, 64, 65].

A prospective cohort study in Tanzania and Uganda examined the retention in care and clinical outcomes of an integrated care model for HIV, DM, and HTN. Integration translated in this case to setting up a single clinic for the management of patients with HIV, DM, or HTN, or a combination of these. Patients shared waiting areas, reception, and pharmacy, and were seen by the same clinical staff. In addition, systems for patient follow-up, counselling, appointments, registration, and recording clinical notes were aligned to streamline disease management regardless of the condition. Healthcare staff also received training in all three conditions to ensure a common understanding. The study reported high retention rates across disease categories, including 82.5% in HIV, 85% in DM, and 78.8% in HTN, and 90.9% among those with multimorbidity. Although blood pressure and blood glucose improved over time in this study, a high percentage of participants with HTN and DM continued to have uncontrolled levels. The authors highlighted that the limited availability of adequate monitoring tests for DM, particularly HbA1c testing, posed a challenge during the study [66]. Three studies in rural South Africa [64], Kenya and Uganda [39], and Malawi [65], respectively, tested an integrated model of care for HIV/AIDS and HTN. For example, the integrated HIV and HTN model of care in Malawi utilized a referral network for individuals with complicated HTN and developed an HTN-specific module in the Malawi electronic medical record system. The results showed that 11% of the population were newly diagnosed with HTN, and 85% of those received treatment. 38% and 30% had controlled mild HTN and moderate HTN, respectively [65]. However, the results of a controlled interrupted time-series study in South Africa, which examined the clinical effectiveness of a model of care, Integrated Chronic Disease Management (ICDM), on participants with HIV, HTN, and DM, indicated a small effect in the control of blood pressure (1% higher likelihood of achieving blood pressure control). The authors outlined that the failure to achieve successful blood pressure control in this setting may be due to health system and individual-related factors [64].

Furthermore, a randomized clinical trial at four socioeconomically diverse clinics in India examined the potential of an integrated coordinated care program for patients with DM and depression. The intervention used team-based, patient-centered approaches to overcome barriers and facilitate patient, clinician, and system-level mechanisms. Care coordinators and consulting specialists, such as psychiatrists and diabetologists, enhanced standard care by supporting diabetes physicians. The results showed an improvement of depressive symptoms, a reduction of at least 0.5 percentage points in HbA1c, and a reduction of at least 5 mm Hg in systolic blood pressure. The authors also highlighted the increased access, feasibility, and acceptability of implementing the integrated collaborative model of care across various DM care settings [47].

#### CMO3: digitalized infrastructure and literacy (C), facilitate mHealth and eHealth implementation and clinical decentralization of DM and/or HTN (M), improving access, coordination, and treatment adherence (O)

Digital development and advancement in technology literacy [45, 60, 62] serve as an enabling context, which facilitates the implementation of mHealth, eHealth, and clinical decentralization of healthcare services. These mechanisms improve access, treatment adherence, and support coordination both among healthcare workers and between healthcare workers and patients [35, 42, 43, 45, 46, 48, 50, 51, 54, 60, 62, 63, 65].

A study in rural settings of Cameroon reported that infrastructure for a telemedicine center, TELEMED-CAM, at one of the two largest general hospitals was established, and specialized physicians were trained to utilize the computer software for the remote monitoring of patients. Healthcare workers in remote centers were also allowed to communicate and collaborate with the telecare center for further guidance on their decision-making and to receive immediate feedback. The study highlighted that the broad availability of mobile phones and the internet in Africa has offered new and affordable means of communication, which facilitated telemedicine as a potential alternative for enhancing access to chronic disease care where human resources are limited. In this study, blood pressure control among patients improved. Among participants with stage III HTN and participants with stage I and II HTN at baseline, 50% and 65.2% in the intervention group achieved the target blood pressure at the final visit (P = 0.20) [60]. Another study in Argentina evaluated an intervention combining a mobile app with a DM registry, a provider-focused clinical decision support system, and a patient-targeted text messaging intervention. The authors noted that mobile technology’s penetration is a significant driver that influences the implementation of digital health interventions. This study showed a significant increase in the percentage of participants undergoing laboratory check-ups and receiving pharmacological treatment. The study also found a decreased rate of uncontrolled blood pressure from 47.2% to 30.8% [62]. Moreover, a study in Lebanon underscored the enhanced patient-provider interactions via the implementation of an mHealth app. The results revealed a higher percentage of lifestyle counseling, medication complications, and follow-up scheduling. Systolic and diastolic blood pressure measurements decreased by 8.4% following the mHealth phase compared with baseline (P = 0.001). The authors also reported that the mHealth app has the potential to enhance adherence to guidelines in Lebanon [63].

#### CMO4: patient-centered care (C) leads to improved patient-provider communication and personalized care (M), increasing patient engagement and health education (O)

Some studies indicated that patient-centered care [41, 45, 46, 49] enhances interpersonal communication between healthcare providers and patients, and personalized care, leading to improved patient engagement and health education [35, 37, 38, 41, 43–46, 49, 50].

In a study conducted in China, a nurse-led HTN management team adopting the Chronic Care Model incorporated home visits, telephone follow-ups, and assistance in improving patients’ self-management and health education. Patients also received a free annual health check. The results showed that the reductions in systolic blood pressure and diastolic blood pressure in the study group compared to the control group were −14.37 mmHg and −7.43 mmHg, respectively and were significant (P = 0.003 and P = 0.002). Patients also had significantly greater improvement in self-care behaviors and a higher level of satisfaction [41]. Similarly, in another study conducted in China, the primary care physician provided health education and medication advice to a patient face-to-face or through text messages. Blood pressure control rates were 47.4% in the intervention group and 30.2% in the control group (P <0 .001). The authors highlighted that the positive results on HTN management are potentially due to the various components being coordinated in a way that improved communication between patients and their primary care physicians, and that telemedicine provided an effective and new way for managing this condition [45].

Personalized care, as part of patient-centered care, allows for customizing patients’ treatment through health technologies, as outlined in two studies. For example, a study conducted in eight Asia-Pacific countries used technology-guided structured evaluation (JADE) to personalize reports to encourage patient empowerment and patient engagement. Nurses were trained to use the JADE technology. The use of information and communications technology and nurses to empower and engage patients reduced cardiometabolic risk factors, such as HbA1c, among patients with T2DM without affecting clinical event rates. The effects were stronger in LMICs [35]. Similarly, a study in India integrated nurse-led care coordination with a mobile phone-based clinical decision support system (mDSS), which was used to create a personalized patient management plan. The results showed 52% of newly detected cases of HTN and 30% of DM. The reductions in systolic blood pressure, diastolic blood pressure, and fasting plasma glucose observed equaled 14.6 mm Hg (95% CI: 15.3, 13.8), 7.6 mm Hg (CI: 8.0, 7.2), and 50.0 mg/dL (95% CI: 54.6, 45.5), respectively and were statistically significant [46].

### Causal explanations of CMO configurations and program theory refinement

**FIGURE 2:**
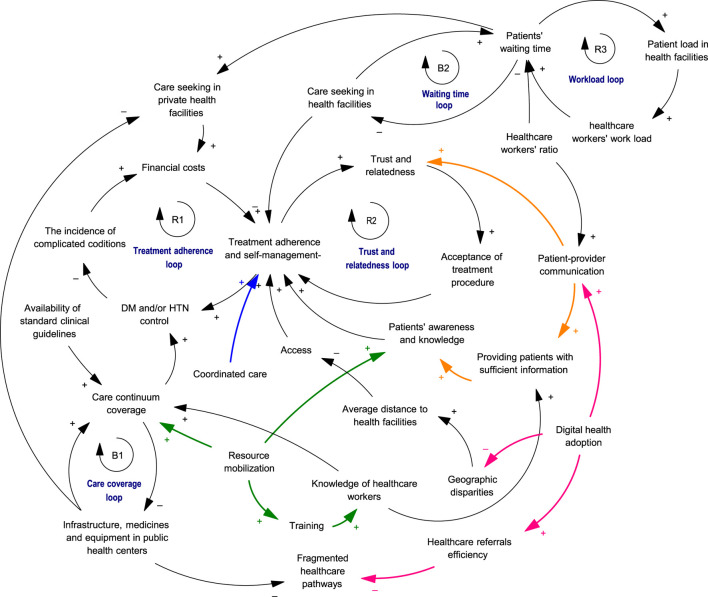
Causal relationships of factors influencing the management of Diabetes Mellitus (DM) and/or Hypertension (HTN). Balancing loops (B), Reinforcing loops (R), the mechanisms of identified configurations (green arrows = related to CMO1, blue arrow = related to CMO2, pink arrows = related to CMO3, orange arrows = related to CMO4) (A realist review to understand the complexity of effective management of type 2 diabetes and hypertension, 2013–2023) (low- and middle-income countries).

The key causal links involved in the management of DM and/or HTN The key causal links involved in the management of DM and/or HTN based on three reinforcing loops and two balancing loops, and the mechanisms of four CMO configurations are presented in an updated CLD (Figure 2).

In the first reinforcing loop (R1), the level of treatment adherence and self-management affects the incidence of complications [67–71] and further contributes to patients’ financial costs [71–75]. Patients’ awareness and knowledge improve treatment adherence and self-management [69, 70, 73, 74]. In contrast, poor awareness leads to complicated conditions as patients may only seek care after complications arise. The delay in diagnosis and treatment adherence increases the risk of severe conditions [76, 77]. However, patient-provider communication and providing patients with sufficient information increase patients’ awareness and knowledge [67–71]. This realist review found that CMO4, through patient-centered care, facilitates patient-provider communication, improving patient engagement and health education [35, 37, 38, 41, 43–46, 49, 50]. Moreover, digital health adoption improves CMO4 [35, 43, 45, 46, 63]. CMO1 also enhances patient awareness and education [56, 61]. Additionally, geographic disparities in many rural and remote areas increase the average distance to health facilities and transportation costs, which negatively influence access [67, 69–71, 74, 76] and further affect patient treatment adherence. However, this realist review found that the implementation of mHealth and telehealth within the CMO3 configuration addresses geographic disparities and improves access [60].

The first balancing loop (B1) illustrates that the availability of infrastructure, medicines, and equipment in public health facilities improves the care continuum coverage, including screening, diagnosis, and treatment [67, 71, 76, 78]. Additionally, adherence to standard clinical guidelines [67, 76] strengthens the level of care continuum coverage. However, insufficient infrastructure, medicines, and equipment in public health facilities increase the fragmentation of healthcare pathways [69, 78], decrease care-seeking in public health facilities [67, 71, 74], and increase care-seeking in private health facilities [73, 74, 78]. Care-seeking in private health facilities increases costs, and patients may reduce their treatment adherence [71, 74, 79]. CMO1, through resource pooling and mobilization within the context of cross-sectoral partnerships, strengthens care continuum coverage [36, 52, 56, 59, 61] and improves the training of healthcare workers [40, 55, 57–59]. This realist review also found that CMO3, through the implementation of digital health, improves structured healthcare referrals and coordination [42, 43, 50, 60, 65].

In the second reinforcing loop (R2), the level of trust and relatedness of patients to the healthcare system influences the acceptance of the disease and treatment, which leads to better treatment adherence and self-management [68]. Patient-provider communication increases trust and relatedness with healthcare workers [67–69, 74, 79]. This realist review found that CMO4 improves the level of patient-provider communication [35, 37, 38, 41, 43–46, 49, 50]. In the second balancing loop (B2), waiting time [70, 71] has a negative effect on care-seeking in health facilities. As a result, it may drive care-seeking in private health facilities [79]. In addition, the third reinforcing loop (R3) shows patient load that intensifies with waiting time [69, 71] as one of the drivers of health workers’ workload [71]. A low healthcare workers ratio also increases the healthcare workers’ workload [76], which influences the level of patient-provider communication and providing patients with sufficient information about their conditions [69, 79]. CMO3, through the mechanisms of implementation of mHealth and eHealth, increases clinical decentralization and task sharing [54], which may reduce health workers’ workload. mHealth also directly improves patient-provider communication [63].

In addition, the enabling CMOs not only illustrate that they are neither independent nor mutually exclusive but also underline the patterns of interdependency and synergistic behavior that trigger changes in the management of DM and/or HTN [35, 42, 43, 45, 46, 51, 52, 65]. For example, we found that digitalized infrastructure and literacy facilitate mechanisms of mHealth and eHealth implementation that enhance patient-provider communication and personalized care, leading to improved patient knowledge and awareness [35, 43, 45, 46].

## Discussion

This realist review identified four overarching CMO configurations of programs targeting DM and/or HTN across various settings in LMICs. The findings show that the contexts comprising digitalized infrastructure and literacy, patient-centered care, integration of disease-specific programs for chronic conditions, and cross-sectoral partnerships moderate the execution of the underlying mechanisms, leading to better health outcomes. This study also examined the key pathways and feedback loops and explored how CMO configurations may influence the management of DM and/or HTN through a CLD, a key tool to operationalize systems thinking.

Digitalized infrastructure and literacy facilitate the mechanisms associated with the implementation of mHealth and eHealth, supporting better access, coordination, and treatment adherence. A realist synthesis in Southeast Asia reported that in rural regions with limited access to healthcare facilities, telehealth has the potential to serve certain healthcare needs of the community, if supported by sufficient technical infrastructure [80]. mHealth interventions could also improve healthcare in patients with DM and support hard-to-reach individuals; however, racial/ethnic minority groups, older adults, and those with limited health literacy or more depressive symptoms demonstrated the lowest engagement in an mHealth intervention, highlighting the potential for socioeconomic disparities in outcomes [81]. Evidence from a systematic review suggests that mHealth interventions are associated with clinical improvements in HbA1c in LMICs [82]. In contrast, a systematic review and meta-analysis in Africa reported inconclusive evidence regarding the effectiveness of mHealth interventions to decrease blood pressure and glycemic control among affected patients [83]. The successful implementation of digital health technologies necessitates the development of supportive policies, stable electricity infrastructures, affordable mobile internet service, an understanding of the socio-economic and sociocultural beliefs, and improvement of technological literacy [84–86].

Our findings revealed that patient engagement and health education improve within the context of patient-centered care, where mechanisms of patient-provider communication and personalized care are triggered. An integrative review of patient-centered care for patients with cardiometabolic diseases indicated that patient-centered care is associated with improvements in factors such as communication, patient-practitioner relationships, shared decision making, medication adherence, engagement and responsibility for health, and self-care and care continuity [87]. Furthermore, a realist review of the management of DM in people with dementia reported that person-centered care triggers mechanisms such as trust, empowerment, and a belief that self-management is achievable and worthwhile [32]. Our findings also suggest that in the context of patient-centered care, digital health synergizes the mechanisms related to patient-provider communication and personalized care, highlighting that CMOs are interrelated and dependent.

Another overarching CMO configuration that emerged from our analysis was that integration of disease-specific programs for chronic conditions enables the mechanisms of coordinated care, resulting in treatment adherence. However, a systematic review and meta-analysis of studies in South Africa, India, Kenya, and Uganda revealed that integrated models of care for DM/or HTN with any other disease may make little or no difference to quality of life, mortality, control of DM and HTN, and evidence on all health outcomes is significantly uncertain [88]. In South Africa, the Integrated Chronic Disease Management (ICDM) for HIV, HTN, and DM also demonstrated only a small clinical effect on the control of blood pressure [64]. This may reflect health system-related contextual limitations in the primary healthcare facilities, including HTN medication stock-outs, malfunctioning blood pressure machines, shortages of healthcare workers, and long patient waiting times [89].

Lastly, a wide range of cross-sectoral partnerships exists between international, national, and local communities and between public and private sectors. Strengthening collaboration across communities through the active involvement of community members and leadership from local, state, and national public and private sectors is essential [90]. In this regard, CARDIO4Cities for HTN in Ulaanbaatar in Mongolia, Dakar in Senegal, and São Paulo in Brazil was facilitated within a context of multisectoral partnerships. Across these cities, common characteristics included increasingly sedentary lifestyles and shifts in dietary patterns [91]. However, it is essential to account for contextual variations that may drive mechanisms and produce different health outcomes across settings. In Dakar, one study exploring the effectiveness of the cascade of care for HTN in the CARDIO4Cities initiative identified both contextual enablers and barriers. Health system-related constraints included a lack of support for community health workers, overburdened healthcare workers, and low motivation. Additional factors, such as patients’ trivialization and denial of HTN, as well as traditional gender roles and the strong impact of low socio-economic status, were reported [92]. Another study in São Paulo, Brazil, examined the complexity of the management of HTN with a systems thinking lens using a CLD. The research revealed several socio-economic constraints, including distance to healthcare facilities, educational level, and family income, and noted that gender, ethnicity, and the availability of private health insurance could affect access to care. These interrelated contextual factors reflect the complexity involved in HTN treatment access [93]. In this review, using a systems thinking lens allowed us to move beyond linear explanations of health system components. The resulting program theory suggests that effective management of DM and/or HTN occurs within complex and interdependent causal pathways and feedback loops.

### Limitations of the review

One key limitation of this review concerns our level of certainty regarding the generalizability of the CMOs to all LMIC settings. Some studies do not explicitly highlight contexts, mechanisms, or the outcomes resulting from the interaction of the contexts and the mechanisms. Therefore, we were unable to extract a comprehensive CMO for these studies. This limitation might arise from insufficient descriptions of CMOs in these studies or other contexts that may contribute to these mechanisms, ultimately influencing the observed outcomes. Additionally, some contexts and mechanisms related to the patients, family, community, and broader socio-cultural dynamics were not in the scope of this review.

### Conclusion

Healthcare services for DM and/or HTN involve complex and interdependent interactions. This realist review provides insights into how beneficial changes in DM and/or HTN management are facilitated by enabling health system-related contextual factors such as digitalized infrastructure and literacy, cross-sectoral partnerships, integration of disease-specific programs for chronic conditions, and patient-centered care. This review suggests designing and implementing effective models of care in LMICs that take into account the interplay of enabling contexts and their triggered mechanisms to address barriers to the management of DM and/or HTN. Moreover, combining a realist approach with a systems thinking lens is recommended for addressing complex issues within health systems.
