# Supplementary material for: A realist review to understand the complexity of effective management of type 2 diabetes and hypertension
Source: Public Health Rev. 2026 Jun 1;47:1608655. doi: 10.3389/phrs.2026.1608655 (PMC13266464; doi:10.3389/phrs.2026.1608655)
Supplement: Supplementary file 1 [file DataSheet2.pdf]

Table S2: Database Search

PubMed search strategy

#	Query
#1	(Health Care Services [mesh] OR Community Health Services[mesh] OR Care, Primary Health[mesh] OR Health Care, Primary[mesh] OR Healthcare, Primary[mesh] OR Primary Care[mesh] OR Care, Primary[mesh] OR Care, Secondary[mesh] OR Secondary Cares[mesh] OR Model of Care[Title/Abstract] OR Care Model[Title/Abstract] OR Chronic Care Model[Title/Abstract] OR Health Care Services [Title/Abstract] OR Community Health Services [Title/Abstract] OR primary care [Title/Abstract] OR Secondary Care [Title/Abstract] OR intervention [Title/Abstract] OR intervention program [Title/Abstract] OR program [Title/Abstract] OR package [Title/Abstract] OR "care model" OR "model of care"))
#2	Diabetes Mellitus, Type 2 [mesh] OR Diabetes Mellitus, Type II [mesh] OR Type 2 Diabetes Mellitus [mesh] OR Type 2 Diabetes [mesh] OR Diabetes, Type 2 [mesh] OR Diabetes Mellitus, Type 2 [Title/Abstract] OR Diabetes Mellitus, Type II [Title/Abstract] OR Type 2 Diabetes [Title/Abstract] OR Type II Diabetes Mellitus [Title/Abstract] OR diabet* Type 2 [Title/Abstract] OR Type 2 diabet* [Title/Abstract] OR diabet* Type II [Title/Abstract] OR "Type 2 diabetes" OR "Diabetes Type 2"
#3	Hypertension [mesh] OR Blood Pressure, High [mesh] OR Blood Pressures, High [mesh] OR High Blood Pressure [mesh] OR High Blood Pressures [mesh] OR High Blood Pressure [Title/Abstract] OR Hypertensi* [Title/Abstract] OR elevated blood pressure [Title/Abstract] OR "high blood pressure" OR "raised blood pressure"
#4	#1 AND #2 AND #3
#5	List of LMICs
#6	4# AND 5#

Web of Science search strategy

(TI=((Health Care Services OR Community Health Services OR Care, Primary Health OR Health Care, Primary OR Healthcare, Primary OR Primary Care OR Care, Primary OR Care, Secondary OR Secondary Cares OR Model of Care OR Care Model OR Chronic Care Model OR intervention OR program OR intervention program Or package) AND (Hypertension OR Blood Pressure, High OR High Blood Pressure OR Hypertensi* OR elevated blood pressure OR raised blood pressure) AND (Diabetes Mellitus, Type 2 OR Diabetes Mellitus, Type II OR Type 2 Diabetes Mellitus OR Type 2 Diabetes OR Diabetes, Type 2 OR diabet* Type 2 OR Type 2 diabet* OR Type II diabet*) AND (list of LMICs)
OR AB=((Health Care Services OR Community Health Services OR Care, Primary Health OR Health Care, Primary OR Healthcare, Primary OR Primary Care OR Care, Primary OR Care, Secondary OR Secondary Cares OR Model of Care OR Care Model OR Chronic Care Model OR intervention OR program OR intervention program Or package) AND (Hypertension OR Blood Pressure, High OR High Blood Pressure OR Hypertensi* OR elevated blood pressure OR raised blood pressure) AND (Diabetes Mellitus, Type 2 OR Diabetes Mellitus, Type II OR Type 2 Diabetes Mellitus OR Type 2 Diabetes OR Diabetes, Type 2 OR diabet* Type 2 OR Type 2 diabet* OR Type II diabet*) AND (list of LMICs)

EMBASE search strategy

('health care services':ab,ti OR 'community health services':ab,ti OR 'care, primary health':ab,ti OR 'health care, primary':ab,ti OR 'healthcare, primary':ab,ti OR 'primary care':ab,ti OR 'care, primary':ab,ti OR 'care, secondary':ab,ti OR 'secondary cares':ab,ti OR 'model of care':ab,ti OR 'care model':ab,ti OR 'chronic care model':ab,ti OR intervention:ab,ti OR program:ab,ti OR 'intervention program':ab,ti OR package:ab,ti) AND (hypertension:ab,ti OR 'blood pressure, high':ab,ti OR 'high blood pressure':ab,ti OR hypertensi*:ab,ti OR 'elevated blood pressure':ab,ti OR 'raised blood pressure':ab,ti) AND ('diabetes mellitus, type 2':ab,ti OR 'diabetes mellitus, type ii':ab,ti OR 'type 2 diabetes mellitus':ab,ti OR 'type 2 diabetes':ab,ti OR 'diabetes, type 2':ab,ti OR 'diabet* type 2':ab,ti OR 'type 2 diabet*':ab,ti OR 'type ii diabet*':ab,ti) AND (list of LMICs)
