# Supplementary material for: A realist review to understand the complexity of effective management of type 2 diabetes and hypertension
Source: Public Health Rev. 2026 Jun 1;47:1608655. doi: 10.3389/phrs.2026.1608655 (PMC13266464; doi:10.3389/phrs.2026.1608655)
Supplement: Supplementary file 2 [file DataSheet4.pdf]

**Quality Appraisal**

| Category of study designs | Methodological quality criteria | Yes | No | Can't tell |
|---|---|---|---|---|
| Screening questions (for all types) | S1. Are there clear research questions? | 32\32 | | |
| | S2. Do the collected data allow to address the research questions? | 32\32 | | |
| Quantitative randomized controlled trials | 2.1. Is randomization appropriately performed? | 10/13 | 0/13 | 3/13 |
| | 2.2. Are the groups comparable at baseline? | 12/13 | 1/13 | 0/13 |
| | 2.3. Are there complete outcome data? | 12/13 | 0/13 | 1/13 |
| | 2.4. Are outcome assessors blinded to the intervention provided? | 3/13 | 4/13 | 6/13 |
| | 2.5 Did the participants adhere to the assigned intervention? | 10/13 | 1/13 | 2/13 |
| Quantitative non-randomized | 3.1. Are the participants representative of the target population? | 8/9 | 1/9 | 0/9 |
| | 3.2. Are measurements appropriate regarding both the outcome and intervention (or exposure)? | 8/9 | 0/9 | 1/9 |
| | 3.3. Are there complete outcome data? | 7/9 | 1/9 | 1/9 |
| | 3.4. Are the confounders accounted for in the design and analysis? | 5/9 | 2/9 | 2/9 |
| | 3.5. During the study period, is the intervention administered (or exposure occurred) as intended? | 7/9 | 0/9 | 2/9 |
| Quantitative descriptive | 4.1. Is the sampling strategy relevant to address the research question? | 2/2 | 0/2 | 0/2 |
| | 4.2. Is the sample representative of the target population? | 2/2 | 0/2 | 0/2 |
| | 4.3. Are the measurements appropriate? | 2/2 | 0/2 | 0/2 |
| | 4.4. Is the risk of nonresponse bias low? | 2/2 | 0/2 | 0/2 |
| | 4.5. Is the statistical analysis appropriate to answer the research question? | 2/2 | 0/2 | 0/2 |
| Mixed methods | 5.1. Is there an adequate rationale for using a mixed methods design to address the research question? | 5/5 | 0/5 | 0/5 |
| | 5.2. Are the different components of the study effectively integrated to answer the research question? | 5/5 | 0/5 | 0/5 |
| | 5.3. Are the outputs of the integration of qualitative and quantitative components adequately interpreted? | 5/5 | 0/5 | 0/5 |
| | 5.4. Are divergences and inconsistencies between quantitative and qualitative results adequately addressed? | 5/5 | 0/5 | 0/5 |
| | 5.5. Do the different components of the study adhere to the quality criteria of each tradition of the methods involved? | 3/5 | 0/5 | 2/5 |

**Description of the results of the quality appraisal**

Most RCTs (n=10) reported appropriate randomization, and eight studies indicated that the groups were comparable at baseline and provided complete outcome data. Ten RCTs reported that participants adhered to the assigned interventions. However, only a small number of studies (n=3) reported that the outcome assessors were blinded to the intervention provided. Among quantitative non-randomized studies (n=9), the majority (n=8) showed that the participants were representative of the target population, and the measurements were appropriate for both the outcomes and interventions. Moreover, most studies (n=7) reported complete outcomes, and some studies (n=5) accounted for confounders in the study design and analysis. While some studies (n=6) reported that the interventions were administered as intended, for three studies, we could not tell whether this was the case. For two out of four quantitative descriptive studies, we identified that their sampling

strategies were relevant to address the research questions. The samples were representative of the target population, the measurements were appropriate with a low risk of nonresponse bias, and their statistical analyses were appropriate for answering the research questions. All mixed-methods studies (n=5) also provided adequate rationale for using a mixed-methods design, effectively integrated the different components to answer the research questions, and adequately interpreted the outputs. No divergences were observed between the qualitative and quantitative findings. However, two studies received a “cannot tell” rating regarding the adherence of individual components to the quality criteria of each tradition of the methods involved.
