# Supplementary material for: A realist review to understand the complexity of effective management of type 2 diabetes and hypertension
Source: Public Health Rev. 2026 Jun 1;47:1608655. doi: 10.3389/phrs.2026.1608655 (PMC13266464; doi:10.3389/phrs.2026.1608655)
Supplement: Supplementary file 3 [file DataSheet3.pdf]

## Characteristics of included studies

| Reference | Country | Year | Study design | condition under study | CMO1 | | | CMO2 | | | CMO3 | | | CMO4 | | |
|---|---|---|---|---|---|---|---|---|---|---|---|---|---|---|---|---|
| | | | | | C | M | O | C | M | O | C | M | O | C | M | O |
| Lim L-L et al. [37] | 8 Asia-Pacific countries (India, Indonesia, Malaysia, the Philippines, Singapore, Taiwan, Thailand, and Vietnam) | 2021 | RCT | DM | | | | | | | | ✓ | ✓ | | ✓ | ✓ |
| Boch J et al. [44] | Ulaanbaatar in Mongolia, Dakar in Senegal, and São Paulo in Brazil | 2022 | Observational implementation study | HTN | ✓ | ✓ | ✓ | | | | | | | | | |
| Ali MK et al. [66] | India and Pakistan | 2016 | RCT | DM | | | | | | | | ✓ | | | ✓ | |
| Jafar TH et al. [67] | Bangladesh, Pakistan, and Sri Lanka | 2016 | A mixed-method feasibility study | HTN | | | | | | | | | | | ✓ | ✓ |
| Hickey MD et al. [51] | Kenya and Uganda | 2021 | RCT | HTN | | | | ✓ | ✓ | ✓ | | | | | | |
| Lou Q et al. [45] | China | 2020 | RCT | DM | ✓ | ✓ | ✓ | | | | | | | | | |
| Zhu X et al. [64] | China | 2021 | RCT | HTN | | | | | | | | | | ✓ | ✓ | ✓ |
| Wang Z et al. [53] | China | 2018 | Observational study (a retrospective study) | HTN and/or DM and COPD | | | | ✓ | ✓ | | | ✓ | ✓ | | | |
| Jia W et al. [61] | China | 2021 | RCT | DM | | | | | | | | ✓ | ✓ | | ✓ | ✓ |
| Sun Y et al. [68] | China | 2022 | RCT | HTN | | | | | | | | | | | ✓ | |
| Zhou H et al. [57] | China | 2022 | RCT | HTN | | | | | | | ✓ | ✓ | ✓ | ✓ | ✓ | ✓ |
| Ajay VS et al. [58] | India | 2016 | Mixed-methods study | DM and HTN | | | | | | | | ✓ | ✓ | ✓ | ✓ | ✓ |

| | | | | | | | | | | | | | | | | |
|---|---|---|---|---|---|---|---|---|---|---|---|---|---|---|---|---|
| Ali MK et al. [52] | India | 2020 | RCT | DM and depression | | | | ✓ | ✓ | ✓ | | | | | | |
| Prabhakaran D et al. [59] | India | 2019 | RCT | HTN and DM | | | | | | | | ✓ | | | | |
| Ramli AS et al. [65] | Malaysia | 2016 | RCT | DM | | | | | | | | | | ✓ | ✓ | ✓ |
| Low W et al. [60] | Malaysia | 2013 | A pragmatic non-RCT | HTN | | | | | | | | ✓ | ✓ | | | |
| Paluyo J et al. [38] | Philippine | 2023 | Observational study | DM and HTN | ✓ | ✓ | ✓ | | | | ✓ | ✓ | ✓ | | | |
| Pilleron S et al. [46] | Philippines | 2014 | Observational study | DM | ✓ | ✓ | ✓ | | | | | | | | ✓ | ✓ |
| Khan MA et al. [47] | Pakistan | 2018 | Mixed-methods study | HTN | ✓ | ✓ | ✓ | | | | | | | | | |
| Xie W et al. [63] | Bangladesh | - | Observational study | DM and HTN | | | | | | | | ✓ | ✓ | | | |
| Chan BT et al. [48] | Kazakhstan | 2020 | A quasi-experimental study | DM and HTN | ✓ | ✓ | ✓ | | | | | | | | | |
| Valdes Gonzalez Y et al. [40] | Cuba | 2020 | Implementation study | HTN | ✓ | ✓ | ✓ | | | | | | | | | |
| Dethlefs HJ et al. [41] | Dominican Republic | 2019 | A quality improvement study | DM and HTN | ✓ | ✓ | ✓ | | | | | | | | | |
| Adler AJ et al. [43] | Ghana | 2019 | Observational study (Cohort) | HTN | ✓ | ✓ | ✓ | | | | | | | | | |
| Collins DR et al. [49] | Tajikistan | 2013 | RCT | HTN | ✓ | ✓ | ✓ | | | | | | | | | |
| Kingue S et al. [56] | Cameroon | 2019 | Prospective interventional study | HTN | | | | | | | ✓ | ✓ | ✓ | | | |
| Zou G et al. [42] | Sierra Leone | 2020 | Mixed-methods study | HTN | ✓ | ✓ | ✓ | | | | | | | | | |
| Morelli DM et al. [55] | Argentina | 2023 | A pragmatic quasi- | DM | | | | | | | ✓ | ✓ | ✓ | | | |

| | | | | | | | | | | | | | | | | |
|---|---|---|---|---|---|---|---|---|---|---|---|---|---|---|---|---|
| | | | experimental study | | | | | | | | | | | | | |
| Doocy S et al. [62] | Lebanon | 2017 | Observational study (Cohort) | DM and HTN | | | | | | | | ✓ | ✓ | | | |
| Ameh S et al. [50] | South Africa | 2017 | A controlled interrupted time-series | HTN, HIV | | | | ✓ | ✓ | ✓ | | | | | | |
| Patel P et al. [39] | Malawi | 2018 | Observational study | HTN and HIV | ✓ | ✓ | ✓ | ✓ | ✓ | ✓ | | ✓ | ✓ | | | |
| Birungi J et al. [54] | Uganda and Tanzania | 2021 | Observational study | HIV, DM and HTN | | | | ✓ | ✓ | ✓ | | | | | | |

RCT=Randomized Controlled Trial, HTN= Hypertension, DM= Diabetes Mellitus, COPD= Chronic Obstructive Pulmonary Disease, C=context, M= mechanism, O= health outcome
