# Supplementary material for: A realist review to understand the complexity of effective management of type 2 diabetes and hypertension
Source: Public Health Rev. 2026 Jun 1;47:1608655. doi: 10.3389/phrs.2026.1608655 (PMC13266464; doi:10.3389/phrs.2026.1608655)

The geographic and income distribution and the number of included studies in each country

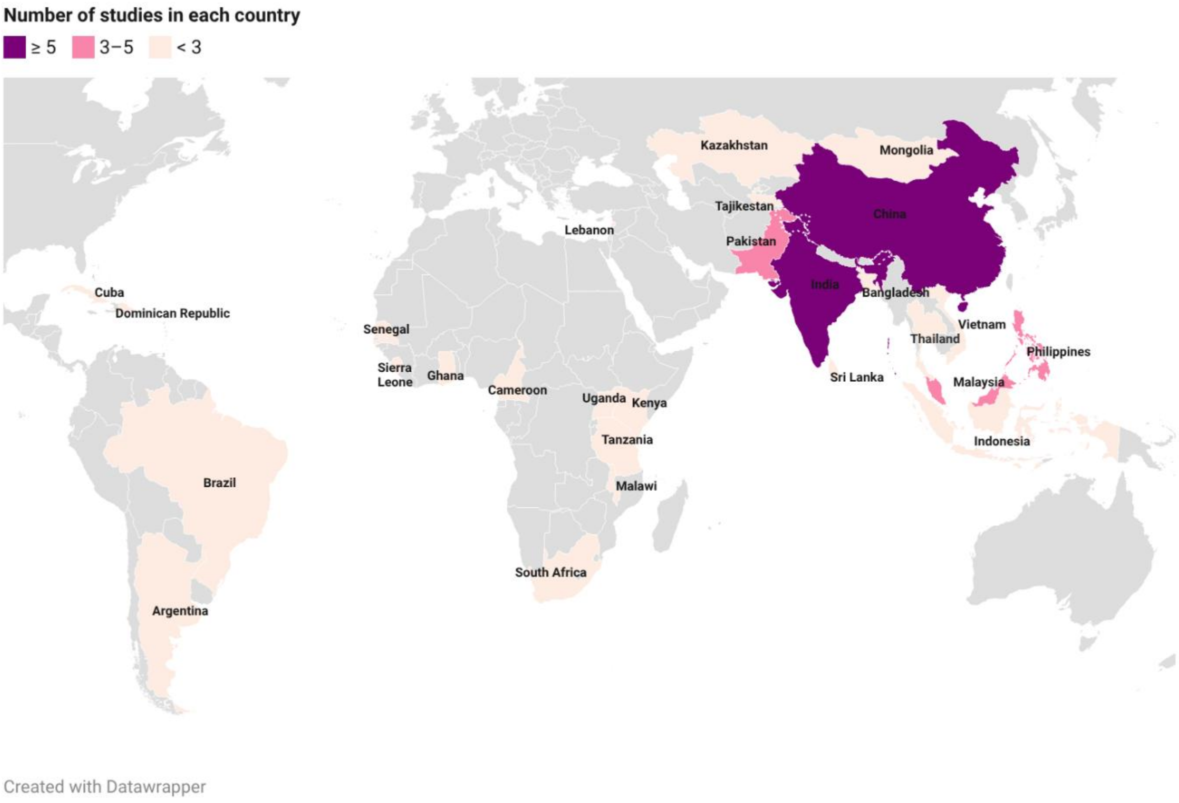

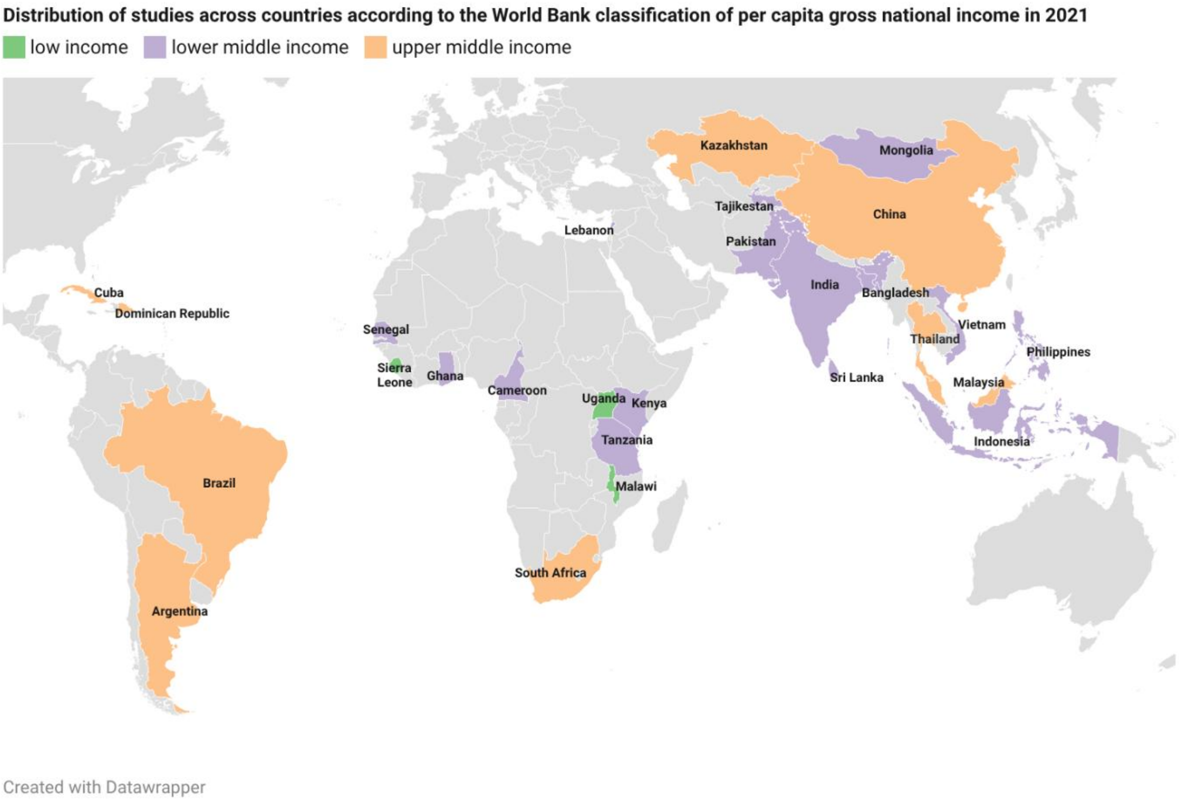

Supplement: Supplementary file 5 [file DataSheet5.pdf]
